# The Role of the Immunological Synapse in Differential Effects of APC Subsets in Alloimmunization to Fresh, Non-stored RBCs

**DOI:** 10.3389/fimmu.2018.02200

**Published:** 2018-10-05

**Authors:** Amanda L. Richards, Kathryn Sheldon, Xiaoping Wu, David R. Gruber, Krystalyn E. Hudson

**Affiliations:** ^1^Bloodworks Northwest Research Institute, Seattle, WA, United States; ^2^Department of Laboratory Medicine, University of Washington School of Medicine, Seattle, WA, United States

**Keywords:** Red blood cells (RBC), transfusion, alloimmunization, inflammation, alloantibodies

## Abstract

**Background:** Each year, over 5 million red blood cell (RBC) transfusions are administered to patients in the USA. Despite the therapeutic benefits of RBC transfusions, there are associated risks. RBC-specific alloantibodies may form in response to antigenic differences between RBC donors and recipients; these alloantibodies can be a problem as they may mediate hemolysis or pose barriers to future transfusion support. While there is currently no reliable way to predict which RBC recipients will make an alloantibody response, risk factors such as inflammation have been shown to correlate with increased rates of RBC alloimmunization. The underlying mechanisms behind how inflammation mediates alloantibody production are incompletely defined.

**Methods:** To assess erythrophagocytosis, mice were treated with PBS or inflammatory stimuli followed by a transfusion of allogeneic RBCs labeled with a lipophilic dye. At multiple time points, RBC consumption and expression of activation makers by leukocytes was evaluated. To determine which antigen presenting cell (APC) subset(s) were capable of promoting allogeneic T cell activation, sorted leukocyte populations (which had participated in erythrophagocytosis) were co-cultured *in vitro* with allogeneic CD4+ T cells; T cell proliferation and ability to form immunological synapses with APCs were determined.

**Results:** Upon transfusion of fresh allogeneic RBCs, multiple APCs consumed transfused RBCs. However, only CD8+ and CD11b+ dendritic cells formed productive immunological synapses with allogeneic T cells and stimulated proliferation. Importantly, allogeneic T cell activation and RBC alloantibody production occurred in response to RBC transfusion alone, and transfusion in the context of inflammation enhanced RBC consumption, the number of immune synapses, allogeneic T cell proliferation, and the rate and magnitude of alloantibody production.

**Conclusions:** These data demonstrate that regardless of the ability to participate in RBC consumption, only a subset of APCs are capable of forming an immune synapse with T cells thereby initiating an alloantibody response. Additionally, these data provide mechanistic insight into RBC alloantibody generation.

## Introduction

Red blood cell (RBC) transfusions are administered to ~1 out of 70 Americans annually. Transfusions are given as supportive care to treat anemia as a result of genetic deficiencies, bone marrow failure or suppression, and blood loss as a consequence of trauma, surgery, chronic bleeding, or childbirth. Whilst transfusions are extremely beneficial for vulnerable populations, they also have risks associated with them. Specifically, exposure to allogeneic RBCs, which express different antigens from the recipient's own RBCs can lead to the generation of RBC-specific alloantibodies (1–3). RBC alloantibodies can be a clinically significant problem as they may pose barriers to future transfusion support, cause adverse transfusion reactions (e.g., hemolysis), and lead to hemolytic disease of the fetus and newborn in women of childbearing age (4–7). For chronically transfused patients (e.g., Sickle Cell Disease, Myelodysplastic Syndrome, etc.), development of alloantibodies to multiple RBC antigens can result in a lack of compatible RBC donor units (8). Thus, chronically transfused patients may suffer morbidity, or in extreme cases mortality, due to lack of sufficient compatible blood.

The underlying factors that regulate alloimmunization are poorly understood. Alloantibody generation occurs in up to 10% of RBC transfusion recipients (excluding ABO and RhD alloimmunization). Currently, there is no reliable way to predict which recipients will make an RBC alloantibody. However, there are risk factors that correlate with RBC alloimmunization, such as genetics, number of transfusions, and inflammatory state of the recipient (9–12). Recently, it has been shown in both murine models and in the human setting that receipt of an RBC transfusion in the presence of inflammation correlates with higher rates of alloimmunization (9, 12–16). However, not all types of inflammation produce this effect; while alloimmunization rates are higher in recipients with viral infections, rates of alloantibody generation are reduced in recipients with gram negative bacterial infections (14, 17–19). As such, the mechanisms that underlie the differential effects of distinct types of inflammation on RBC alloimmunization are of significant interest.

Murine models with defined model antigens on RBCs have been useful in efforts to understand the relationship between inflammation and subsequent alloimmunization. Using such models, it has been reported that RBC transfusion into mice treated with poly (I:C), a synthetic double-stranded RNA agonist that mimics a viral-like inflammation, leads to increased rates of alloimmunization whereas treatment with lipopolysaccharide (LPS), cell membrane components derived from gram-negative bacteria, suppresses alloantibodies (17). Additional mechanistic analyses have identified dendritic cells (DCs) to be of critical importance for this process; however, the DC subtype responsible remains contentious. Our group demonstrated that poly (I:C)-elicited inflammation shifts syngeneic RBC consumption to immunostimulatory antigen presenting cells (APCs) such as plasmacytoid DCs (pDCs) (20). A different study demonstrated that poly (I:C) acted on CD8+ DCs to induce type 1 interferon production, which was required for RBC alloantibody generation (21). In contrast, a third group provided evidence that CD8+ DCs are dispensable for alloimmunization to transfused stored RBCs; instead, CD11b+ DCs are required for presentation of RBC antigens to CD4 T cells and subsequent alloantibody production (22). Although different DC subsets were implicated in each study, it should be noted that the use of stored RBCs *per se*, likely represents an inherent source of inflammation (with a response distinct from that of viral or bacterial mediators), composed of oxidized RBC proteins, free iron, and hemoglobin (23). Thus, taken together, different types of inflammation may dictate subsequent RBC alloimmunization by acting on distinct APC subsets.

Herein we utilized the HOD murine model of allogeneic RBC transfusion to gain mechanistic insight as to how poly (I:C)-elicited inflammation leads to enhanced alloimmunization by evaluating RBC clearance, APC consumption patterns, and allogeneic T cell activation. HOD transgenic mice express an RBC-restricted triple fusion protein consisting of *h*en egg lysozyme, *o*valbumin, and human blood group molecule *D*uffy (HOD) (24). In particular, we show that allogenic HOD RBC clearance from circulation is not affected by poly (I:C)-elicited inflammation. Whilst no gross changes were observed in the periphery, at early time points, inflammation led to increased erythrophagocytosis by monocytes, pDCs, and neutrophils in the spleen. Through co-culture experiments, we show that at baseline and under inflammatory conditions, CD8+ and CD11b+ DC subsets presented RBC-derived antigens, formed productive immunological synapses with T cells, and drove T cell proliferation. In contrast, monocytes, pDCs, and neutrophils failed to induce T cell proliferation. Thus, taken together, these data indicate that while multiple APC subsets play a role in RBC consumption at baseline and during inflammation, conventional DC subsets are essential for inducing T cell activation thereby regulating RBC alloimmunization.

## Materials and methods

### Mice

C57Bl/6 (B6) mice were purchased from Charles River Laboratories. HOD transgenic mice were generated as previously described (24) and are bred at Bloodworks NW. B6.Cg-Tg(TcraTcrb)425Cbn/J (OTII; stock #004194) mice were purchased from Jackson Labs. Mice were maintained on standard rodent chow and water in a light- and temperature-controlled environment. B6 and HOD mice were used at 8–12 weeks of age and all procedures were performed according to approved Institutional Animal Care and Use Committee (IACUC) protocols.

### Lipophilic dye labeling of murine red blood cells (RBCs)

RBCs from donor B6 and HOD mice were obtained through cardiac puncture and collected in CPDA-1. RBCs were leukoreduced (Acrodisc WBC filter, Pall Life Sciences), washed with sterile PBS, then labeled with DiO (3,3′-dihexadecyloxacarbocyanine perchlorate, Molecular Probes Invitrogen, Carlsbad, CA, USA) or DiI (chloromethylbenzamido 1,1′-dioctadecyl-3,3,3′,3′-tetramethylindocarbocyanine perchlorate, Molecular Probes, Invitrogen) as previously described (25). In brief, HOD RBCs were resuspended in PBS to a 10% hematocrit and 100 uL of DiO (12.5 ug) was added for each 10 mL of RBCs. After labeling at 37°C for 30 min, RBCs were washed 3 times with PBS, and a sample was analyzed by flow cytometry to confirm labeling (Supplementary Figure 1). DiO fluorescence was read on the FL-1 (FITC) channel. B6 RBCs were diluted 1:14 in PBS (total volume 15 mL) and stained with 100 uL (50 ug) of DiI. B6 RBCs were incubated for 18 min at 37°C followed by another 12 min at room temperature, washed 3 times with PBS, and an aliquot was run on a flow cytometer to confirm labeling. DiI fluorescence was read on the FL-2 (PE) channel.

### Treatment of mice and RBC transfusion

For RBC clearance studies, RBCs were harvested from donor mice and leukoreduced. HOD RBCs were labeled with DiO whereas B6 RBCs were labeled with DiI. Recipient mice were transfused with 100 uL of a 1:1 mixture of HOD and B6 RBCs (thus, 50 uL HOD-DiO + 50 uL B6-DiI), and resuspended to a 20% hematocrit in PBS for transfusion. Mice were bled at indicated time points to determine clearance and alloantibody production. In experiments that analyzed RBC consumption, recipient B6 mice were treated with an intraperitoneal injection of 200 ug of Poly (I:C) (GE Healthcare) or sterile PBS 2–4 h prior to transfusion of 100 uL of leukoreduced DiO-labeled HOD RBCs, diluted in a total volume of 500 uL with PBS. Recipient mice were sacrificed at multiple time points and spleens were harvested for analysis.

### Splenocyte harvest and staining

Spleens were harvested using aseptic technique and collected into sterile complete RPMI, collagenase digested, and filtered as previously described (20). Liberated leukocytes were washed with FACS buffer [PBS + 0.2 mg/mL bovine serum albumin (Sigma) + 4 mL/L 0.5 M EDTA (Sigma)] and RBCs were lysed. Leukocytes were treated with Fc block (anti-mouse CD16/32; BD Biosciences) followed by incubation with cell surface antibodies. Following the surface stain, cells were washed with PBS then stained with fixable viability dye (ThermoFisher Scientific) to determine cellular integrity. For OTII *in vitro* co-culture experiments, OTIIs were enriched with a CD4 kit (Miltenyi) and labeled with final concentration of 5 uM CFSE-far red (CFSE-FR). Typical purity of post-enrichment of OTIIs was ~85%.

### *In vitro* co-culture, fixation, and imaging immunological synapses

In some experiments, leukocytes were harvested from B6 recipients that received PBS or poly (I:C) treatment followed by a HOD RBC transfusion. Leukocytes were then stained with antibodies to delineate antigen presenting cell subsets and DiO+ cells (an indirect measure of RBC consumption) were sorted with a FACS Aria. Sorted individual APCs were co-cultured at a 10:1 ratio with enriched CD4+ OTII T cells at a concentration of no more than 1 × 10^6^/mL in complete RPMI and placed in a 37°C, 5% CO_2_ incubator. OTII CD4+ T cells cultured in media alone served as a negative control whereas stimulation with PMA (10 ng/mL) plus ionomycin (1 ug/mL) was a positive control for proliferation assays. In some experiments, co-culture were harvested after 3 days and CFSE-FR dilution was assessed in CD4+Thy1.1+Va2+Vb5.1/5.2+ OTII T cells. For immune synapse imaging experiments, co-cultures of APCs:OTIIs were harvested after 2 days and processed for staining as previously described (26). Briefly, supernatants of each co-culture was aspirated, briefly vortexed, and fixed with 1 mL of 4% paraformaldehyde for 15 min at room temperature. Cells were then transferred to a 1.5 mL eppendorf tube, centrifuged and washed 3 times with PBS. Cells were stained with antibodies against CD11c, F480, Ly6G, and I-Ab (MHCII) to identify APC subset, Va2, CD4, and Thy1.1 to identify OTIIs and CD18 to demark the immune synapse. Cell images were acquired on an ImageStreamX MkII cytometer (Amnis Corporation). 20,000 events per file were recorded twice for each sample using INSPIRE software at 60x magnification with laser powers of 488 nm set at 20 mw, 405 nm at 10 mw, 561 nm at 20 mw and 642 nm at 45 mw. Analysis was performed using IDEAS 6.2 software. Compensation was calculated using single color controls acquired with side scatter and bright field off.

### Antibody detection

Sera from experimental mice was used for flow crossmatch to determine alloantibody production as previously described (27). The following secondary reagents conjugated to the fluorophore APC were utilized: anti-mouse Igs, goat anti-mouse IgG1, goat anti-mouse IgG2b, goat anti-mouse IgG2c, and goat anti-mouse IgG3 (BD Biosciences).

### Antibodies and flow cytometry

Antibodies to Thy1.2, Thy1.1, TER119, CD19, CD11c, CD11b, CD8, F4/80, Ly6C, PDCA1, Ly6G, CD115, NK1.1, CD49b, MHCII (I-Ab), Vb5.1/5.2, Va2, CD4, and CD18 were purchased from ThermoFisher Scientific. Anti-CD86 was purchased from BD Biosciences. All antibodies were directly conjugated. All staining was performed in FACS buffer on ice. Cell analysis was performed on an LSR-II (BD Biosciences) and data analyzed with FlowJo software (Treestar); cell sorting was performed on a FACSARIA (BDBiosciences).

### Statistics

For experiments with 2 groups, a Student's T-test was performed. For time course experiments, a two-way ANOVA with a multiple comparisons test was utilized to determine significance. Significance was set at *p* ≤ 0.05. To indicate significance, ^****^p ≤ 0.0001, ^***^p ≤ 0.001, ^**^p ≤ 0.01, ^*^p ≤ 0.05.

## Results

### Poly (I:C)-elicited inflammation enhances anti-HOD alloantibody production

**Figure 1:**
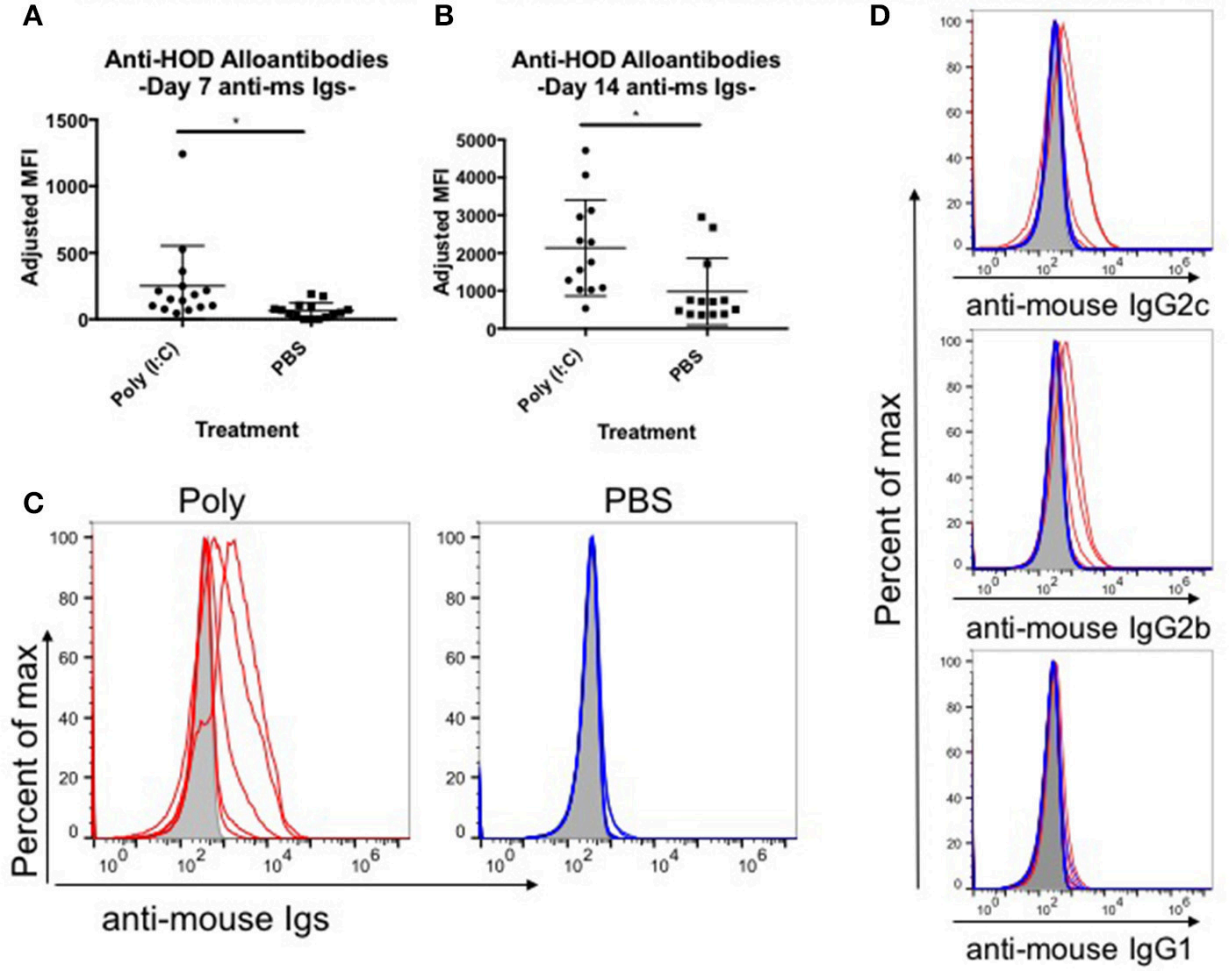
Poly (I:C)-elicited inflammation enhances the magnitude and rate of alloimmunization to HOD RBCs. Recipient B6 mice were treated i.p. with 200 ug of poly (I:C) or PBS control 2–4 h prior to a transfusion of 100 uL packed leukoreduced HOD RBCs. Sera was collected and assessed for anti-HOD alloantibodies by flow crossmatch against HOD RBC targets. The adjusted mean fluorescence intensity (MFI) of anti-mouse Igs APC secondary staining reagent was determined for days **(A)** 7 and **(B)** 14 post RBC transfusion. **(C)** Reactivity of day 14 serum to HOD RBC targets from individual mice in each group is shown by flow crossmatch against HOD RBC targets with total anti-mouse Igs and **(D)** subtype specific IgG secondary reagents. Naïve B6 sera incubated with HOD RBC targets is shown in shaded gray, poly (I:C) treated mice in red, and PBS treated animals in blue; each line represents an individual mouse. This experiment was repeated 3 times with 3–5 mice per group and all experiments are shown. Sera was used at 1:100 dilution. ^*^p ≤ 0.05.

Red blood cell (RBC) transfusion in the context of inflammation can lead to increased RBC alloimmunization. In several described murine models, inflammation with poly (I:C) leads to enhanced RBC alloantibody production (9). To gain more insight into the relationship between inflammation and alloantibody production, we utilized a murine model of transfusion, the HOD mouse. To determine the kinetics, rate, and magnitude of anti-HOD alloantibody production, C57BL/6 (B6) mice were treated with poly (I:C) or PBS control and subsequently transfused with leukoreduced HOD RBCs. Sera was collected at days 7 and 14 and evaluated for anti-HOD alloantibodies by flow crossmatch against HOD and control B6 RBCs. Whereas PBS treated mice had low to undetectable levels of anti-HOD alloantibodies by 7 days post HOD RBC transfusion, mice treated with poly (I:C) had significantly more alloantibodies, as determined by the geometrical mean fluorescence intensity (MFI) of the anti-mouse Igs APC secondary reagent [average MFI: poly (I:C) 251 and PBS 64] (Figure 1A). Similar results were seen at day 14 between poly (I:C)-treated and PBS control mice (Figure 1B). In addition to the higher magnitude of anti-HOD alloantibodies detectable upon inflammation, the number of mice responding also increased upon poly (I:C) treatment (Figures 1B,C). Alloantibody subtype analysis revealed that most anti-HOD alloantibodies generated were IgG2c and IgG2b, with very little IgG1 and low to undetectable levels of IgG3 (Figure 1D and data not shown). Thus, akin to several other murine models of RBC alloimmunization, poly (I:C) enhances both the rate and magnitude of RBC alloantibody production to the RBC-specific HOD alloantigen (9).

### RBC clearance rates are not altered upon inflammation

**Figure 2:**
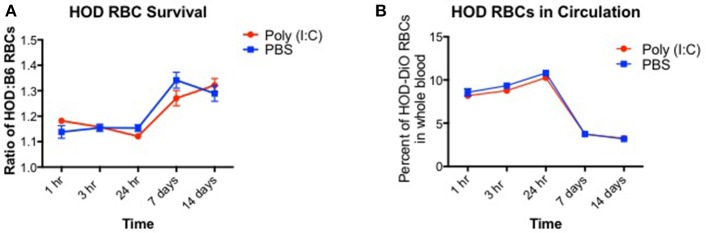
RBC clearance rates are similar between baseline and inflamed conditions. Recipient B6 mice were treated with 200 ug of poly (I:C) or control PBS and subsequently transfused with a 100 uL of 1:1 mixture of DiI+ B6 and DiO+ HOD RBCs. Whole blood was collected at multiple time points and **(A)** the ratio of DiO+ HOD RBCs to DiI+ B6 RBCs was determined to assess allogeneic RBC survival and **(B)** the overall percentage of DiO+ HOD RBCs was determined. Lines: poly (I:C) are red circles, PBS are blue squares. This experiment was repeated 3 times with 3–5 mice per group with similar results. A representative experiment is shown.

Induction of alloantibodies against transfused RBCs may depend on the clearance rate and the length of time that the recipient immune system is exposed to the RBC alloantigen(s). When RBCs are damaged with oxidative agents or heat, the clearance rates are accelerated, and alloantibodies are increased (28). However, if RBCs are damaged too severely such that upon transfusion, they are immediately cleared, only low levels of alloantibodies are detected. Given that poly (I:C)-elicited inflammation leads to enhanced alloimmunization, we hypothesized that HOD RBCs are cleared more quickly in inflamed recipients. To test this hypothesis, recipient B6 mice were treated with poly (I:C) or control PBS and subsequently transfused with a 1:1 mixture of B6 and HOD RBCs, labeled with DiI and DiO lipophilic dyes, respectively (as shown in Supplementary Figures 1A,B). Control DiI+ B6 RBCs were utilized for tracking purposes to determine the rate at which allogeneic HOD RBCs were cleared from circulation over time, as previously described (29). At multiple time points post RBC transfusion, recipient mice were bled and the percentage of circulating DiI+ B6 and DiO+ allogeneic HOD RBCs was determined (as shown in Supplementary Figure 1C). At early time points (hours 1, 3, and 24), DiO+ HOD RBCs were detected in circulation at a similar frequency as control DiI+ B6 RBCs in both PBS and poly (I:C)-treated groups, as demonstrated by a relative survival rate of ~110% (Figure 2A, formula for RBC survival rate shown in Supplementary Figure 1D). However, at 7 and 14 days, detection of DiO+ HOD RBCs was significantly increased over control DiI+ B6 RBCs, as indicated by a higher ratio of HOD:B6 RBCs, suggesting that B6 RBCs were cleared from circulation more quickly than allogeneic HOD RBCs at later time points. Allogeneic HOD RBCs were cleared from circulation, however, as the percentage of DiO+ RBCs of whole blood decreased over time (Figure 2B). Notably, there was no significant difference in the survival of DiO+ HOD RBCs in poly (I:C)-treated mice, compared to control PBS. Thus, taken together, these data reject the hypothesis that poly (I:C)-elicited inflammation significantly altered the detectable circulating frequency of transfused RBCs and suggest that other pathways and/or mechanisms are required for the observed enhanced alloimmunization.

### Effects of poly (I:C) treatment on antigen presenting cells are observed up to 7 days

**Figure 3:**
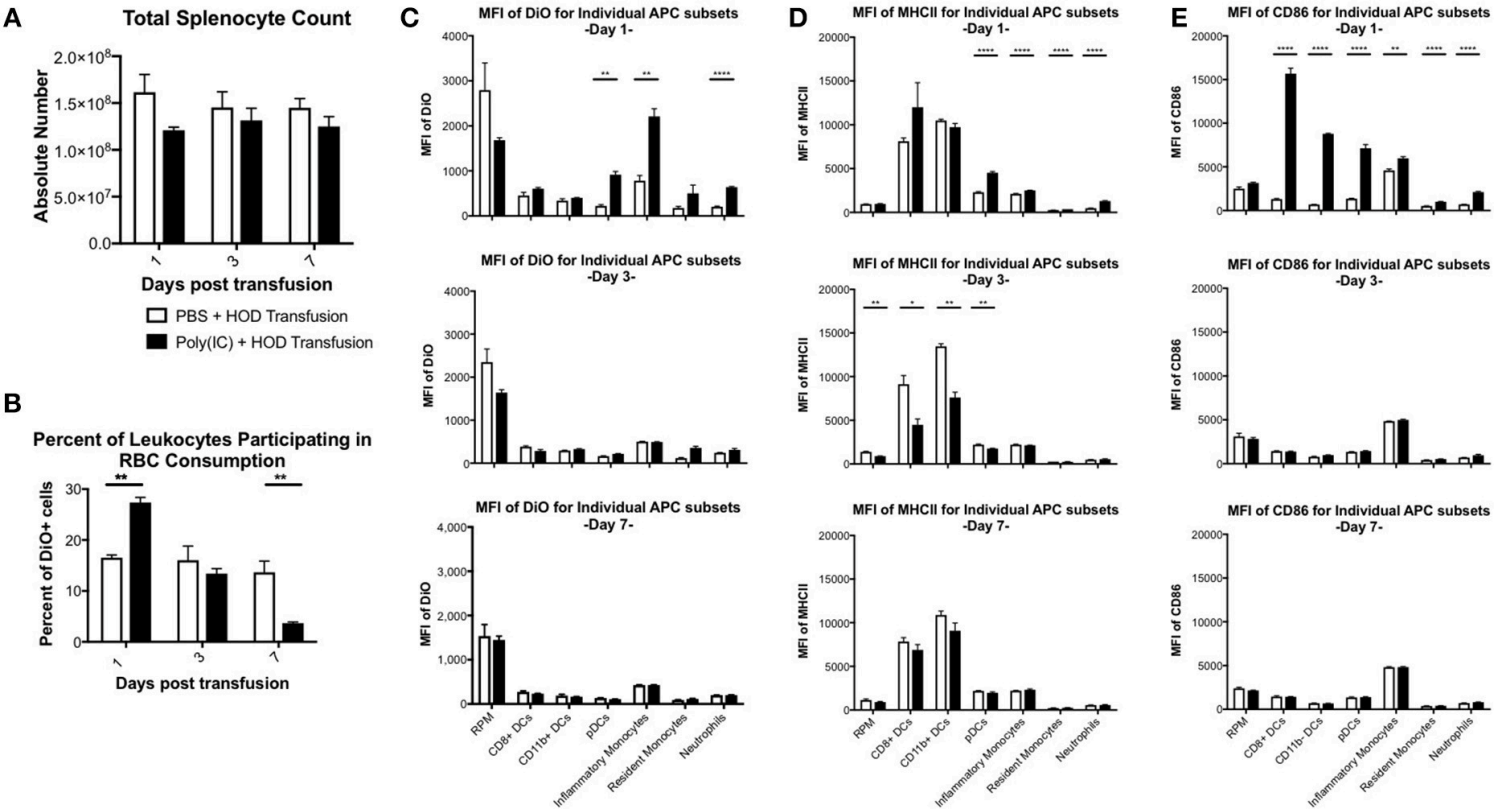
Poly (I:C) leads to increased RBC consumption and upregulation of MHCII and CD86 expression. Recipient B6 mice were treated with poly (I:C) or control PBS and subsequently transfused with 100 uL of packed, leukoreduced, DiO-labeled HOD RBCs. At multiple time points, spleens were harvested, collagenase digested, and stained with antibodies to delineate APC subsets. **(A)** Total splenocyte counts were calculated and **(B)** the percent of DiO+ leukocytes was determined. The MFI of **(C)** DiO, **(D)** MHCII, and **(E)** CD86 was assessed for individual APC subsets. These experiments were repeated 3 times with 3 mice per group with similar results. A representative experiment is shown. PBS treated animals are shown with an open bar and poly (I:C) treatment is shown with a solid black bar. For analysis, T cells, B cells, and RBCs were excluded from total live leukocytes by gating out cells positive for Thy1.2, CD19, NK1.1, CD49b, and TER119. The following phenotypes were used to delineate APC subsets: RPMs: CD11c^−/lo^CD11b^−/lo^F4/80^+^; CD8+ DCs: CD11c^hi^CD11b^−^CD8^+^; CD11b+ DCs: CD11c^hi^CD11b^+^ CD8^−^; pDCs: PDCA1^+^CD11c^int^Ly6C^hi^; inflammatory monocytes: CD11c^−/lo^CD11b^+^Ly6G^−^Ly6C^hi^CD115^+^; resident monocytes: CD11c^−/lo^CD11b^+^Ly6G^−^ Ly6C^lo^CD115^−^; neutrophils: CD11c^−/lo^CD11b^+^Ly6G^+^ and a high side scatter. For significance, ^****^p ≤ 0.0001, ^***^p ≤ 0.001, ^**^p ≤ 0.01, ^*^p ≤ 0.05.

Previous studies have shown that inflammation induced by poly (I:C) has profound effects on splenocyte composition and short-term erythrophagocytosis of transfused syngeneic RBCs (20). Whilst these studies identify monocytes and plasmacytoid dendritic cells (DCs) as antigen presenting cell (APC) subsets that significantly increase RBC consumption under inflammatory conditions, these experiments were limited and could not identify which leukocytes were required for initiation of RBC-specific alloantibody production; utilization of the HOD mice remedies this limitation. Given that HOD RBCs express an antigenic difference from control, syngeneic B6 RBCs, we could not assume that erythrophagocytosis of allogeneic and syngeneic RBCs was identical. To evaluate which APCs participate in RBC consumption and initiate an immune response, recipient B6 mice were treated with poly (I:C) or PBS control followed by a HOD RBC transfusion. Recipient mice were sacrificed at multiple time points and splenocytes were evaluated. Given that the observed population effects of poly (I:C) on APCs in the spleen were visible up to 7 days post inflammation in previous studies (20) and humoral differences were divergent within 1 week (see Figure 1A), we focused on early time points after RBC transfusion. As previously observed, there was no significant difference in overall splenocyte count between treatment groups over time (Figure 3A). However, treatment with poly (I:C) significantly altered the rate of erythrophagocytosis by splenocytes (Figure 3B, representative flow plots shown in Supplementary Figure 2). By as early as day 1 post treatment, the percentage of DiO+ leukocytes, whereby DiO indicates RBC consumption, was significantly increased in poly (I:C)-treated mice, compared to PBS controls (*p* ≤ 0.01). However, by day 3, RBC consumption was similar between experimental and control groups. By day 7, there were significantly fewer leukocytes participating in RBC consumption in poly (I:C)-treated animals, compared to mice given PBS (*p* ≤ 0.01).

Individual APCs were interrogated for DiO content over time. As previously observed, red pulp macrophages (RPMs) consumed the most RBCs at baseline at every time point evaluated, as determined by DiO MFI (Figure 3C, representative histograms shown in Supplementary Figure 3) (20). However, RPMs, unlike every other APC subset analyzed, had a slightly lower DiO MFI upon poly (I:C) treatment, suggesting that fewer RPMs were consuming RBCs during an inflammatory environment. In contrast, RBC consumption was significantly increased in pDCs, inflammatory monocytes, and neutrophils at day 1 (*p* ≤ 0.01, *p* ≤ 0.01, and *p* ≤ 0.0001, respectively), a pattern previously observed with syngeneic erythrophagocytosis (20); however, these observed enhancements were absent at days 3 and 7. Indeed, the RBC consumption patterns at days 3 and 7 were similar between treatment groups for each individual APC subset. Given that allogeneic RBC consumption patterns resembled those previously reported with syngeneic RBC transfusion at baseline and during poly (I:C)-elicited inflammation, these data suggest that an antigenic difference between syngeneic and allogeneic RBCs did not affect which APC subsets participated in erythrophagocytosis.

RBC consumption is not alone sufficient to initiate humoral alloimmunization. For CD4 T cell activation to occur, the T cell must recognize a peptide:MHCII complex and receive co-stimulation. In an inflammatory environment, several APC subsets are induced into maturation and upregulate cell surface expression of both MHCII and co-stimulatory molecules to facilitate interactions with CD4 T cells, inducing activation and subsequent immune responses. To assess which APC subset(s) may be capable of providing these positive, activating signals to responding T cells, each population was evaluated for surface expression of MHCII and CD86. Upon RBC transfusion in the absence of inflammation, no significant modulation of MHCII expression was documented over the time period analyzed. Conventional DCs (cDCs), which include both CD8+ and CD11b+ DC subsets, had the highest level of MHCII expression at baseline. Poly (I:C) treatment led to significant upregulation of MHCII on pDCs, monocytes, and neutrophils in poly (I:C) treated mice by day 1, compared to PBS groups (*p* ≤ 0.0001, Figure 3D, representative histograms shown in Supplementary Figure 3). No significant difference of MHCII MFI was observed between treatment groups in RPMs or cDCs. By day 3, MHCII was significantly downregulated in RPMs, CD8+ DCs, CD11b+ DCs, and pDCs (Figure 3D, middle panel). These findings are consistent with previous reports that post activation, APCs downregulate MHCII (30). By day 7, expression of MHCII between treatment groups was similar (Figure 3D, bottom panel). These data demonstrate that transfusion alone does not modulate expression of MHCII. Additionally, these illustrate the consequences of exposure to the inflammatory agent poly (I:C) are still evident 3 days post treatment.

The pattern of co-stimulatory molecule CD86 expression differed from MHCII; poly (I:C)-elicited inflammation led to significant upregulation of CD86 expression at day 1 by every APC subset evaluated, except RPMs, compared to PBS treated animals (Figure 3E, representative histograms shown in Supplementary Figure 3). Notably, DC subsets (CD8+, CD11b+, and pDCs) had the greatest increase in CD86, which correlates with the maturation of DCs into professional APCs. At later time points, the MFI of CD86 in poly (I:C) treated groups were similar to PBS controls, indicating that the effects of the poly (I:C) inflammatory stimuli were immediate and short-lived. No modulation of CD86 expression on individual APC subsets was observed in PBS-treated mice over the 7 days analyzed. These data demonstrate that transfusion of allogeneic HOD RBCs did not elicit CD86 expression on APC subsets, which indicates that transfusions of fresh, non-stored RBCs, are not inflammatory. Taken together, these data suggest that the cumulative effects of poly (I:C) inflammation (i.e., increased alloantibodies) are the result of innate immune activation; in particular, within 24 h post treatment with poly (I:C) and transfusion, specific APCs increase RBC consumption and upregulate expression of MHCII and CD86.

### HOD RBC-containing cDCs induce proliferation of CD4+ OTII T cells

**Figure 4:**
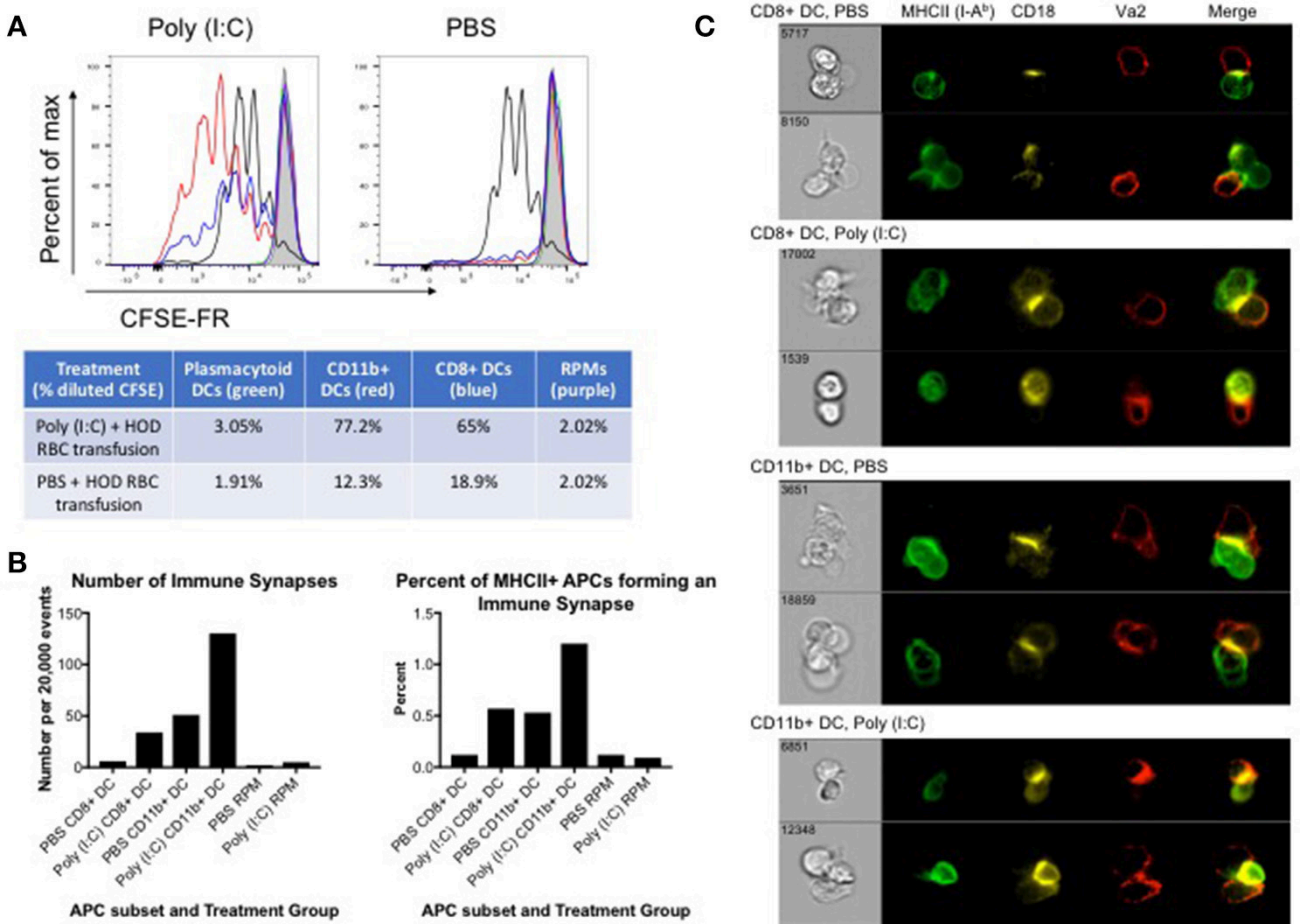
CD8+ and CD11b+ DCs promote proliferation and immune synapses with HOD RBC-specific T cells. Recipient B6 mice were treated with 200 ug of poly (I:C) or control PBS and subsequently transfused with 100 uL of leukoreduced, packed, DiO-labeled HOD RBCs. Spleens were harvested 18–24 h post transfusion, collagenase digested, and stained with antibodies to delineate individual APC populations. DiO+ APCs were sorted and co-cultured at a 10:1 ratio with CD4 enriched OTII T cells labeled with CFSE-FR. **(A)** Co-cultured cells were harvested after 3 days and CFSE-FR dilution was assessed in Va2+Vb5.1/5.2+CD4+Thy1.1+ OTII T cells. Lines: pDCs (green), CD11b+ DCs (red), CD8+ DCs (blue), RPM (purple), PMA/ionomycin (black), and media alone (gray). In separate experiments, co-cultured cells were harvested after 2 days and stained with antibodies to identify APCs, OTII T cells, and the immunological synapse. APCs were identified as MHCII+ (I-A^b^+) and either CD11c+ (for DCs) or F4/80+ (for RPMs) whereas OTIIs were defined as Va2+CD4+Thy1.1+. The immunological synapse was determined by co-expression of Va2, CD4, MHCII, and CD18 (also known as LFA-1). **(B)** The number and frequency of immunological synapses was determined and **(C)** representative images shown. These experiments were repeated 3 times with similar results; a representative experiment is shown.

Many APC subsets, such as DCs, consume transfused allogeneic RBCs and express cell surface molecules known to enhance T cell function and initiate immune responses. To evaluate which APC subsets have the capacity to promote T cell activation, B6 recipients were treated with poly (I:C) or control PBS and transfused with leukoreduced DiO-labeled HOD RBCs. Spleens were harvested 18–24 h post transfusion and individual DiO+ APC subsets were sorted. Individual APC subsets were co-cultured *in vitro* with CFSE-FR-labeled CD4+ OTII T cells. At day 3 post co-culture, cells were harvested and stained with antibodies against Va2, Vb5.1, Thy1.1, and CD4 to identify the OTII CD4+ T cells. OTIIs proliferated robustly (as indicated by decreased signal in CFSE-FR) in the presence of the positive control, PMA/ionomycin (black line) whereas OTIIs did not have diluted CFSE when incubated with media alone (shaded gray) (Figure 4A). In general, APCs isolated from poly (I:C) inflamed animals promoted more OTII proliferation when compared to those treated with PBS. More specifically, both CD11b+ (red line) and CD8+ (blue line) DC subsets drove proliferation of OTIIs. Unexpectedly, despite increased consumption of HOD RBCs and upregulation of co-stimulatory markers upon inflammation, pDCs (green line) did not facilitate OTII proliferation in either treatment group. Likewise, although a predicted observation, RPMs (purple line) did not lead to OTII T cell proliferation. Similarly, no OTII division was observed in co-cultures with monocytes or neutrophils (data not shown). In co-cultures with CD11b+ DCs, the percentage of OTIIs with diluted CFSE was 77.2% in response to poly (I:C) and 12.3% in the PBS group. Similarly, CD8+ DCs from poly (I:C) treated animals prompted 65% of OTIIs to proliferate, compared to 18.9% when the DCs were harvested from spleens of PBS treated mice. Taken together, these data demonstrate that both CD8+ and CD11b+ DC subsets are capable of promoting RBC-specific OTII T cell proliferation. Importantly, these data show that OTII T cell proliferation can occur in mice treated with PBS, representative of a baseline response, and poly (I:C)-elicited inflammation only enhances the effect on CD4+ T cells.

To evaluate whether individual APC subsets formed productive immunological synapses with CD4+ OTIIs, sorted DiO+ APCs were co-cultured *in vitro* with CD4+ OTIIs. After 2 days, co-cultured cells were harvested, fixed with paraformaldehyde, stained with antibodies against cell surface molecules known to be contained within the immunological synapse, and analyzed with an Amnis imaging flow cytometer. APCs were identified by expression of MHCII and either CD11c (for DCs) or F4/80 (RPMs) whereas OTIIs were defined as CD4+Va2+Thy1.1. The formation of an immunological synapse was determined by co-localization of Va2, CD4, MHCII (I-A^b^) [all 3 molecules located within the central supramolecular activation complex (cSMAC)] and shared expression of CD18, also known as LFA-1, located within the peripheral supramolecular activation complex (pSMAC) (31). Gating strategy is provided in Supplementary Figure 4 and images from the immune synapse region were evaluated. Immunological synapses were enumerated and a percentage of synapses of total MHCII-expressing cells was calculated (Figure 4B). Both CD8+ and CD11b+ DC subsets formed immunological synapses with co-cultured OTIIs (Figure 4C, representative images). Consistent with *in vitro* proliferation data, CD11b+ DCs formed more synapses than CD8+ DCs and more synapses were observed with DCs harvested from spleens of mice treated with poly (I:C) compared to PBS control (Figure 4B). This observation is consistent with published data that CD11b+ DCs are more efficient at activating CD4 T cells. Few events were detected in the immune synapse region in OTII co-cultures with RPMs; RPMs did not form a significant number of synapses (on average, 2–5 immune synapses per 20,000 events depending on treatment conditions) with co-cultured OTIIs (Figure 4B and Supplementary Figure 5), suggesting that these APCs, upon RBC consumption, may not express sufficient co-stimulatory or co-inhibitory markers to promote successful T cell interactions. Similar to co-cultures with RPMs, a small number of events were detected in the immune synpase region in OTIIs stimulated with PMA/Ion or cultured in media alone. However, upon evaluation of the imaged cells, no immune synapses were detected as, despite a positive MHCII signal, the levels were either low and close to background noise or were antibody particulates (Supplementary Figure 5). This demonstrates that this protocol distinguishes between proliferating cells and cell-to-cell contact. Thus, taken together, these data provide evidence of direct cell-to-cell contact and immunological synapse formation between cDCs and OTIIs, which is correlated with enhanced OTII proliferation and subsequent anti-HOD alloantibody production.

## Discussion

Generation of alloantibodies to RBCs can be a clinically significant problem. One factor that influences alloimmunization is transfusion in the context of inflammation. Herein, we confirm that RBC transfusion in the context of poly (I:C)-elicited inflammation enhances alloantibody production. We further show that poly (I:C) leads to significant increases in erythrophagocytosis and upregulation of surface expression of MHCII and CD86 by multiple splenic APC subsets at early time points. Whilst many APC subsets respond to inflammatory signals, only CD8+ and CD11b+ DCs are capable of stimulating allogeneic CD4+ OTII T cell proliferation and forming productive immune synapses. Thus, taken together, these data demonstrate that cDCs are essential in the initiation of an alloimmune response to allogeneic RBC transfusions.

One mechanism by which inflammation may enhance alloimmunization is through modulating the clearance rate of transfused RBCs. Our data show that poly (I:C)-elicited inflammation had no effect on the survival or percentage of allogeneic HOD RBCs detectable in the circulation, when compared to PBS treated mice. Thus, these data suggest that alloimmunization may be due to subtle changes (likely occurring in tissues) not detectable in the periphery. This rationale is further supported by data demonstrating that poly (I:C) inflammation leads to a significant increase of DiO+ leukocytes in the spleen within 24 h post-treatment. As such, these data provide additional evidence that clearance rate (as detectable in the periphery) may not be a reliable predictor of whether alloantibodies will be generated upon RBC transfusion.

We have previously shown that poly (I:C)-elicited inflammation results in an increase of erythrophagocytosis of syngeneic RBCs by inflammatory monocytes and pDCs (20). The data presented herein build on those observations by testing whether patterns of RBC consumption are altered in response to allogeneic RBCs. These studies provide evidence that both syngeneic and allogeneic RBC consumption are performed by the same APC subsets at baseline and during poly (I:C) inflammation; RPMs consume the most RBCs whereas inflammation leads to an increase of RBC consumption by inflammatory monocytes and pDCs. These experiments also identify neutrophils as a cell subset that significantly increases erythrophagocytosis upon inflammation; our previous studies did not evaluate the role of neutrophils in RBC consumption.

These data also extend the time frame of analysis to include up to 7 days post treatment and reveal that poly (I:C) effects are still evident beyond what is typically considered an innate immune response. Despite similar frequencies of HOD RBCs in circulation, inflammation led to a significant increase of RBC consumption at early time points but a decrease at day 7. This observation could be due to the relative increase in absolute numbers of certain cell subsets that partake in RBC consumption upon inflammation followed by decreased erythrophagocytosis and downregulation of MHCII post activation. Importantly, however, the immediate effects of poly (I:C) are evident in multiple APCs subsets known to promote T cell activation. Consistent with previous studies, immunostimulatory DC subsets increase expression of MHCII and CD86, molecules that facilitate interactions with T cells. Taken together, it is likely that multiple APC subsets coordinate-not only through direct T cell interactions but also through soluble cytokine secretions to promote activation of allogeneic T cells.

Whilst many APC subsets participate in RBC consumption, only CD8+ and CD11b+ DCs are capable of driving allogeneic T cells to proliferate *in vitro*. Unexpectedly, DiO+ cDCs from non-inflamed mice formed productive immune synapses with OTIIs and led to proliferation. Importantly, these studies utilize fresh RBCs, not stored, thereby eliminating potential inflammatory signals as a result of the storage lesion (32). Thus, these data provide further evidence that allogeneic RBC transfusion, in the absence of inflammation, is capable of stimulating helper T cell activation and promoting low levels of alloantibody production. Importantly, given that transfusion alone does not lead to enhanced expression of MHCII or co-stimulatory CD86, the mechanisms by which activating signals are promoted remain unclear. What is clear, however, is that inflammatory signals due to poly (I:C) administration effectively convert a weak immune response into a strong one through enhanced RBC consumption, upregulation of MHCII and CD86, increased numbers of immune synapse formation and T cell proliferation. The cumulative effect of these changes is a significant increase in RBC alloantibodies. Thus, these data provide additional insight into how inflammation amplifies a low level immune response to enhance alloimmunization.

In contrast to our observations with cDCs, RPMs did not promote allogeneic T cell proliferation. At both baseline and inflammatory states, RPMs consumed the most RBCs among any APC subsets, which is consistent with previous observations (14). However, RPMs failed to promote *in vitro* OTII proliferation. Moreover, no immune synapses were detected upon imaging (Supplementary Figure 5). Thus, in line with previous findings, RPMs likely do not play a role in presentation of RBC-derived antigens (14, 22). However, we cannot rule out that the formation of immune synapses did occur between RPMs and OTIIs but with different kinetics as immune synapses do not always lead to T cell proliferation due to competition within the cSMAC between co-activating and co-inhibitory molecules (33). As such, it is a balance of activating and inhibitory signals that ultimately shape the subsequent immune response. However, of all the images acquired, the RPMs had very few cells with detectable MHCII (data not shown); thus, it is plausible that RPMs do not express enough surface molecules required for an immune synapse to form. It must also be noted that the ability to drive T cell proliferation *in vitro* may not reflect *in vivo* biology. As such, the contributions of each cell subset *in vivo* should be an additional line of future investigation.

The studies presented herein are restricted to the analysis of splenocyte erythrophagocytosis. Previous work by Hendrickson et al. showed that, in addition to splenic leukocytes, CD11c^+^ DCs, and F4/80^+^ macrophages in the liver also participate in erythrophagocytosis (17). However, the same study shows that RBC consumption by leukocytes in the liver is insufficient to drive RBC alloimmunization as no alloantibodies are detectable in splenectomized mice. These data generated with a murine model are consistent with observations in humans whereby splenectomy decreases the risk of RBC alloimmunization (34, 35). However, in some observational studies of thalassemia patients, RBC alloimmunization risk increases upon splenectomy (36, 37). Thus, while the current studies are focused on splenic leukocytes, they were designed to follow-up on previous observations and will eventually be extended to analyze the contribution of liver APCs.

A limitation to the interpretation of the herein rests on the utilization of poly (I:C), a TLR3 agonist, to mimic a viral-like infection. It should be noted that data generated with poly (I:C)-elicited inflammation might not reflect biologies as a result of other agonists (e.g., TLR4) (9, 17, 22). Moreover, not all leukocytes express TLR3; notably, there is no detectable TLR expression in pDCs (38, 39). Thus, in these experiments, activation of non-TLR3 expressing cells may be achieved through poly (I:C) by indirect means—type I interferon and inflammatory cytokine release from cells that express TLR3 (40). Additionally, it has been shown that Type I interferons can act directly on RBCs to induce phosphatidylserine expression, which could lead to increased erythrophagocytosis. As such, poly (I:C) may act on both APCs and RBCs to shape the RBC-specific immune response. Moreover, given that inflammatory stimuli are diverse, follow-up studies should include additional ligands for pathogen pattern recognition receptors to gain a better understanding of how agonists influence RBC alloimmunization.

In summary, we report that allogeneic HOD RBC transfusion can lead to allogeneic T cell proliferation and low levels of alloantibody production. In the context of poly (I:C)-elicited inflammation, however, alloimmunization responses are enhanced. This enhancement is due to increased RBC consumption, upregulation of antigen presenting and co-stimulatory molecules, and increased APC:T cell interactions. Taken together, these data further emphasize the importance of both CD8+ and CD11b+ DC populations in prompting allogeneic T cell proliferations and provide potential mechanisms into how inflammation enhances alloimmunization.

## Ethics statement

All procedures were performed according to protocols approved by the Bloodworks Northwest Institutional Animal Care and Use Committee (IACUC).

## Author contributions

AR, XW, and KH contributed to the conception and design of the study. AR, KS, XW, DG, and KH performed experiments and analyzed data. KH drafted the manuscript with input from all authors.

### Conflict of interest statement

The authors declare that the research was conducted in the absence of any commercial or financial relationships that could be construed as a potential conflict of interest.

## Supplementary material

Supplementary Figure 1 RBC labeling and calculation of RBC survival. RBCs were labeled with lipophilic dyes DiI or DiO and the **(A)** labeling efficiency was determined by flow cytometry. **(B)** RBCs were mixed at a 1:1 ratio and pre-transfusion ratios were determined and **(C)** the relative survival of HOD-DiO RBCs was determined at multiple time points post-transfusion into poly (I:C) and PBS-treated mice (representative blood sampling at day 1 post-transfusion is shown) and overall RBC clearance was calculated with the **(D)** mathematical formula to determine RBC survival in peripheral whole blood.

Supplementary Figure 2 Poly (I:C) treatment affects the percentage of DiO+ leukocytes over time. Recipient B6 mice were treated with poly (I:C) or control PBS and subsequently transfused with 100 uL of packed, leukoreduced, DiO-labeled HOD RBCs. At multiple time points, spleens were harvested, collagenase digested, and stained with antibodies to delineate APC subsets. For determination of the percentage of DiO+ leukocytes, T cells, B cells, and RBCs were excluded from total live cells by gating out cells positive for Thy1.2, CD19, NK1.1, CD49b, and TER119.

Supplementary Figure 3 Poly (I:C) treatment modulates RBC consumption and expression of MHCII and CD86 in RPMs and DC subsets. Recipient B6 mice were treated with poly (I:C) or control PBS and subsequently transfused with 100 uL of packed, leukoreduced, DiO-labeled HOD RBCs. At multiple time points, spleens were harvested, collagenase digested, and stained with antibodies to delineate APC subsets, as shown in Figure 3. Representative data for DiO, MHCII, and CD86 are shown for RPMs, CD8+ DCs, CD11b+ DCs, and pDCs. Lines: PBS (blue) and poly (I:C) (red).

Supplementary Figure 4 Gating strategy to detect immunological synapses. Focused cells are first gated based on Gradient RMS_M01. Cells were then gated on Area_M01 vs. Aspect Ratio_M01 to exclude debris and include both single and aggregated cells. Double positive sells are then sequentially plotted on intensity_MC_M06 (anti-Thy1.1-PE-Cy7) vs. intensity_MC_M02 (anti-I-A^b^-FITC), intensity_MC_M05 (anti-Va2-PerCP-Cy5.5) vs. intensity_MC_M07 (anti-CD11c/F4-80/BV421), and intensity_MC_M03 (anti-CD18-PE) vs. intensity_MC_M11 (CFSE-FR). The all positive cells were further analyzed by Feature Finder in the software. Two parameters Bright detail intensity_R3_MC_CH03 and H contrast mean_M02_CH02 were used to distinguish synapsed vs. non-synapsed cells. The region of synapsed cells have lower H contrast mean of channel 2 (FITC). The non-synapsed cells, which are mainly T cells attached with anti-I-A^b^+ debris on their surfaces, have higher H contrast mean at channel 2. The synapsed cells are further confirmed individually for the presence of CD18 signal at the interface of MHCII+ (I-A^b^+) cells and T cells.

Supplementary Figure 5 No immunological synapse formation between RPMs, media alone, or PMA/Ion treatment. Recipient B6 mice were treated with 200 ug of poly (I:C) or control PBS and subsequently transfused with 100 uL of leukoreduced, packed, DiO-labeled HOD RBCs. Spleens were harvested 18–24 h post transfusion, collagenase digested, and stained with antibodies to delineate individual APC populations. DiO+ CD11c^−/lo^CD11b^−/lo^F4/80^+^ RPMs were sorted and co-cultured at a 10:1 ratio with CD4 enriched OTII T cells labeled with CFSE-FR. OTIIs were stimulated with PMA/Ion for a positive control or in media alone for a negative control. After 2 days in culture, cells were harvested and stained to identify immune synapses. The immunological synapse was determined by co-expression of Va2, CD4, MHCII, and CD18 (also known as LFA-1). Images are from the immune synapse region based on the gating strategy in Supplementary Figure 4. Images are representative from 3 independent experiments.
