# Supplementary figures and images for: The Role of the Immunological Synapse in Differential Effects of APC Subsets in Alloimmunization to Fresh, Non-stored RBCs
Source: Front Immunol. 2018 Oct 5;9:2200. doi: 10.3389/fimmu.2018.02200 (PMC6182098; doi:10.3389/fimmu.2018.02200)

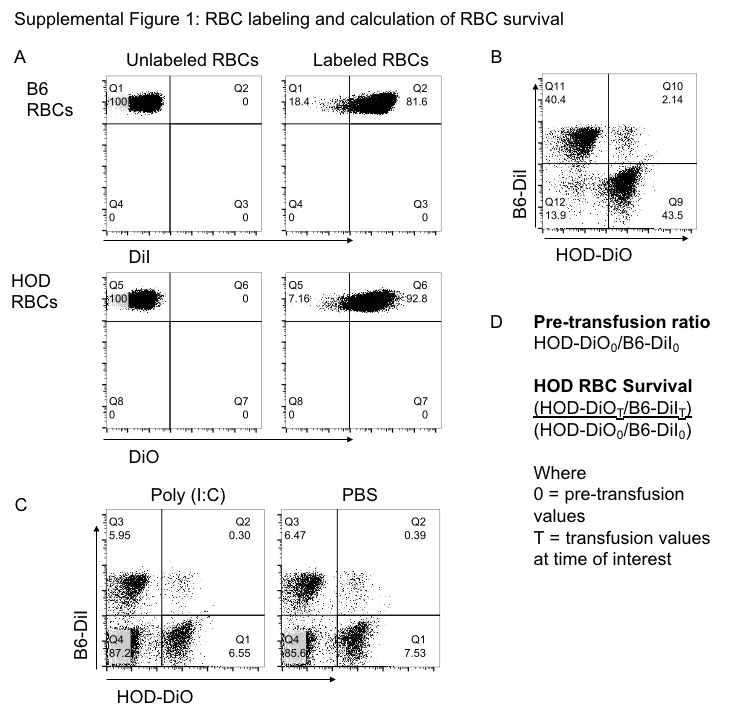

Supplement: Supplementary Figure 1 — RBC labeling and calculation of RBC survival. RBCs were labeled with lipophilic dyes DiI or DiO and the (A) labeling efficiency was determined by flow cytometry. (B) RBCs were mixed at a 1:1 ratio and pre-transfusion ratios were determined and (C) the relative survival of HOD-DiO RBCs was determined at multiple time points post-transfusion into poly (I:C) and PBS-treated mice (representative blood sampling at day 1 post-transfusion is shown) and overall RBC clearance was calculated with the (D) mathematical formula to determine RBC survival in peripheral whole blood. [file Image_1.jpg]

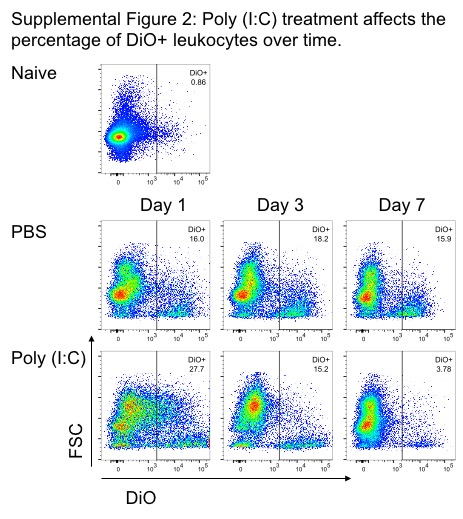

Supplement: Supplementary Figure 2 — Poly (I:C) treatment affects the percentage of DiO+ leukocytes over time. Recipient B6 mice were treated with poly (I:C) or control PBS and subsequently transfused with 100 uL of packed, leukoreduced, DiO-labeled HOD RBCs. At multiple time points, spleens were harvested, collagenase digested, and stained with antibodies to delineate APC subsets. For determination of the percentage of DiO+ leukocytes, T cells, B cells, and RBCs were excluded from total live cells by gating out cells positive for Thy1.2, CD19, NK1.1, CD49b, and TER119. [file Image_2.jpg]

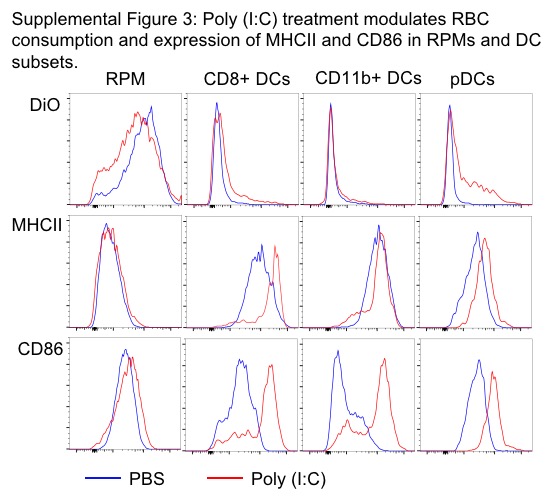

Supplement: Supplementary Figure 3 — Poly (I:C) treatment modulates RBC consumption and expression of MHCII and CD86 in RPMs and DC subsets. Recipient B6 mice were treated with poly (I:C) or control PBS and subsequently transfused with 100 uL of packed, leukoreduced, DiO-labeled HOD RBCs. At multiple time points, spleens were harvested, collagenase digested, and stained with antibodies to delineate APC subsets, as shown in Figure 3. Representative data for DiO, MHCII, and CD86 are shown for RPMs, CD8+ DCs, CD11b+ DCs, and pDCs. Lines: PBS (blue) and poly (I:C) (red). [file Image_3.jpg]

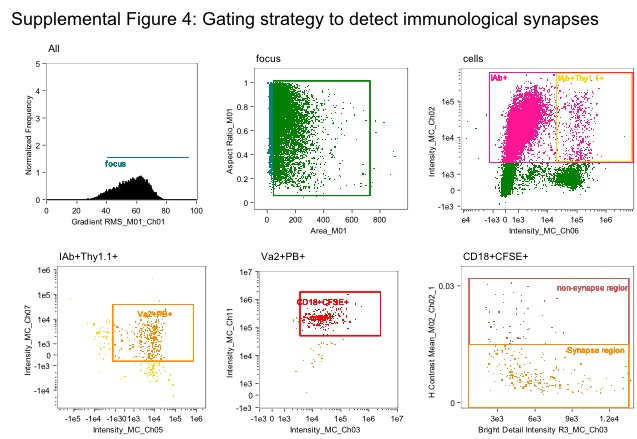

Supplement: Supplementary Figure 4 — Gating strategy to detect immunological synapses. Focused cells are first gated based on Gradient RMS_M01. Cells were then gated on Area_M01 vs. Aspect Ratio_M01 to exclude debris and include both single and aggregated cells. Double positive sells are then sequentially plotted on intensity_MC_M06 (anti-Thy1.1-PE-Cy7) vs. intensity_MC_M02 (anti-I-Ab-FITC), intensity_MC_M05 (anti-Va2-PerCP-Cy5.5) vs. intensity_MC_M07 (anti-CD11c/F4-80/BV421), and intensity_MC_M03 (anti-CD18-PE) vs. intensity_MC_M11 (CFSE-FR). The all positive cells were further analyzed by Feature Finder in the software. Two parameters Bright detail intensity_R3_MC_CH03 and H contrast mean_M02_CH02 were used to distinguish synapsed vs. non-synapsed cells. The region of synapsed cells have lower H contrast mean of channel 2 (FITC). The non-synapsed cells, which are mainly T cells attached with anti-I-Ab+ debris on their surfaces, have higher H contrast mean at channel 2. The synapsed cells are further confirmed individually for the presence of CD18 signal at the interface of MHCII+ (I-Ab+) cells and T cells. [file Image_4.jpg]

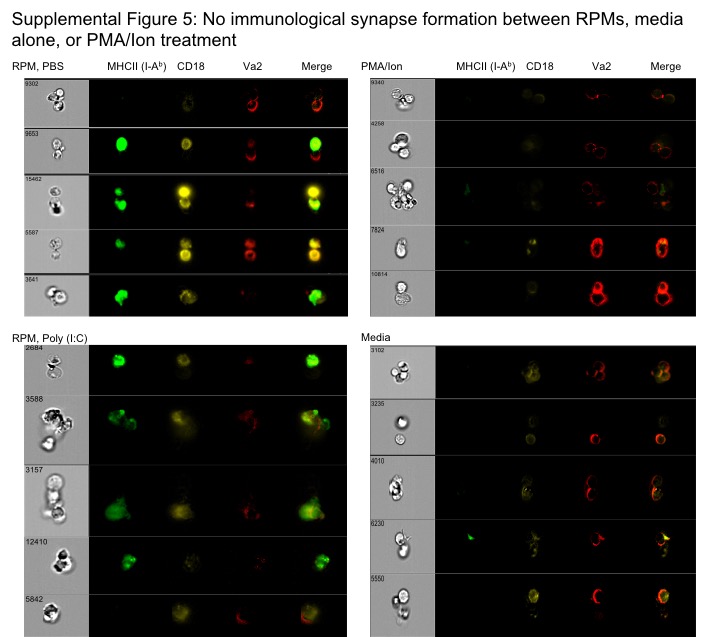

Supplement: Supplementary Figure 5 — No immunological synapse formation between RPMs, media alone, or PMA/Ion treatment. Recipient B6 mice were treated with 200 ug of poly (I:C) or control PBS and subsequently transfused with 100 uL of leukoreduced, packed, DiO-labeled HOD RBCs. Spleens were harvested 18–24 h post transfusion, collagenase digested, and stained with antibodies to delineate individual APC populations. DiO+ CD11c−/loCD11b−/loF4/80+ RPMs were sorted and co-cultured at a 10:1 ratio with CD4 enriched OTII T cells labeled with CFSE-FR. OTIIs were stimulated with PMA/Ion for a positive control or in media alone for a negative control. After 2 days in culture, cells were harvested and stained to identify immune synapses. The immunological synapse was determined by co-expression of Va2, CD4, MHCII, and CD18 (also known as LFA-1). Images are from the immune synapse region based on the gating strategy in Supplementary Figure 4. Images are representative from 3 independent experiments. [file Image_5.jpg]
